# The Role of Japan’s “Hello Baby” Home Visit Program: Secondary Analysis of Municipal Evaluation Data

**DOI:** 10.2196/97276

**Published:** 2026-07-03

**Authors:** Takehiro Arai, Aya Goto

**Affiliations:** 1 Center for Integrated Sciences and Humanities, Fukushima Medical University, Hikarigaoka 1, Fukushima City, 960-1295, Japan, 81 24 547 1835; 2 Faculty of Education, Yamagata University, Yamagata City, Japan; 3 Department of Global Health and Population, Harvard T.H. Chan School of Public Health, Boston, MA, United States

**Keywords:** parenting, loneliness, home visit, social networking, infant, Japan

## Abstract

Given growing concern about maternal loneliness in Japan, this secondary analysis of municipal evaluation data suggests that the nationwide Universal Home Visit Program for Families with Infants (“Hello Baby”) may serve as an initial point of contact and an entry point for expanding parenting support networks, with most mothers receiving a visit and reporting encouragement to talk with someone afterward.

## Introduction

Under Japan’s national policy for child health and development, established in February 2021, the proportion of parents who wish to raise their children in their local community is used as a performance indicator. The policy aims to foster communities that collectively nurture and safeguard children’s healthy development. Maternal loneliness and isolation are key concerns, and support systems are needed to help mothers build local connections [1,2]. Loneliness scales have been applied to parents raising children [3], and social prescribing has been proposed as a policy response to connect mothers with supportive people and resources in their communities [4]. Universal, early-contact programs that reach almost all families may therefore play an important role.

The home visit program for families with infants, known as the Konnichiwa Akachan (Hello Baby) home visit program, was established under the Child Welfare Act in 2009 and has since been implemented nationwide by municipalities. The program visits households with infants up to 4 months of age to listen to parents’ concerns, provide parenting information, assess family needs, and connect families to appropriate services. In Fukushima City, public health nurses and community volunteers each conduct one home visit per infant, and the program is evaluated annually [5]. In this report, we analyze the most recent program evaluation data to examine parents’ willingness to share what they learned from Hello Baby home visits with others as a form of proactive networking.

## Methods

### Study Design

The Hello Baby program evaluation data were obtained from an anonymous survey conducted by Fukushima City in February 2024. The survey targeted households in Fukushima City with children born between April 1 and July 31, 2023. Invitations to participate were mailed to eligible households, and responses were collected through an online system.

### Survey Items

The main survey items were whether respondents received a visit from a health care professional (yes/no); whether they received a visit from a community support volunteer (yes/no); the appropriateness of the timing of the visit (too early, too late, or just right); the degree to which they obtained information about the local area (5-point Likert scale); the degree to which the materials provided were useful (5-point Likert scale); whether the visit prompted them to talk with someone about parenting (yes/no); and whom they spoke with (multiple responses permitted). To assess feelings about receiving the visit, respondents were asked to rate their agreement with each of the following 4 statements: “I felt listened to by the visitor,” “My anxiety was reduced,” “I gained confidence in parenting,” and “I would welcome being approached by a community support volunteer in my local area.” Likert scale responses (“strongly agree,” “agree,” “neither agree nor disagree,” “disagree,” and “strongly disagree”) were dichotomized by combining the first two categories as “Agree” and the remaining three categories as “Do not agree.”

### Statistical Analysis

We first conducted a descriptive analysis of whether the home visit prompted respondents to talk with someone and with whom as our major outcome proxy indicator of proactive networking. Then, we examined the associations between the main outcome indicator and their feelings about receiving the visit by using the *χ*² test. Statistical analyses were conducted using SPSS (version 28), and a *P* value of <.01 was considered statistically significant.

### Ethical Considerations

This study involved a secondary analysis of anonymized data, and it did not fall under the Japanese Ethical Guidelines for Medical Research Involving Human Subjects [6]. Ethical review by Fukushima Medical University was therefore not required.

## Results

Of the 449 households eligible for the program, responses were obtained from 173 (response rate: 38.5%). A total of 94.2% of respondents received a visit from a health care professional, and 91.9% received a visit from a community support volunteer. Overall, 69.6% of respondents reported that the visit prompted them to talk with someone (Table 1). The most common conversation partners were their husband (68.2%), their mother (38.2%), and their friend(s) (17.9%).

Reporting that the visit prompted them to talk with someone was significantly associated with perceiving the timing of the visit as appropriate, finding the materials provided to be useful, and obtaining information about the local area. Regarding respondents’ feelings about the visit, feeling listened to by the visitor, experiencing reduced parenting anxiety, gaining confidence in parenting, and welcoming being approached by a known community support volunteer in the local area were all significantly associated with talking with someone as a result of the visit (Table 2).

**Table 1.** Summary of Hello Baby home visit characteristics (N=173).

	Participants, n	Percentage
Visit from a health care professional		
Received	163	94.2
Not received	10	5.8
Visit from a community support volunteer		
Received	159	91.9
Not received (telephone contact only)	14	8.1
Timing of visit		
Too late	1	0.6
Just right	151	87.3
Too early	18	10.6
Did the visit prompt you to talk with someone?		
Yes	119	69.6
No	52	30.4

**Table 2.** Factors associated with being prompted to talk with someone by the Hello Baby home visit.

	Did the visit prompt you to talk with someone?				
	Yes (N=119)		No (N=52)		*P* value^a^
	Number	Percentage	Number	Percentage	
Timing of the visit					
Too early or too late	9	40.9	13	59.1	.002
Just right	110	73.8	39	26.2	
The materials provided were useful					
Do not agree	7	20.6	27	79.4	<.001
Agree	112	81.8	25	18.2	
I felt listened to by the visitor					
Do not agree	11	42.3	15	57.7	.001
Agree	108	74.5	37	25.5	
I obtained information about the local area					
Do not agree	8	34.8	15	65.2	<.001
Agree	111	75	37	25	
My anxiety was reduced					
Do not agree	17	35.4	31	64.6	<.001
Agree	102	82.9	21	17.1	
I gained confidence in parenting					
Do not agree	23	41.1	33	58.9	<.001
Agree	96	83.5	19	16.5	
I would welcome being approached by a community support volunteer in my local area					
Do not agree	5	23.8	16	76.2	<.001
Agree	114	76	36	24	

a The* χ*² test was used.

## Discussion

Our findings suggest that Japan’s Hello Baby home visit program might serve as an opportunity for parents of infants to engage with others in their community. Similar effects of home visiting have been reported in international reviews [7]. Expanding social networks is a core need for mothers, and it is crucial that they realize they do not have to undertake child-rearing entirely on their own [8]. Beginning to talk with others about parenting can be considered a positive sign of recognizing the existing social network. Maternal loneliness is known to be exacerbated by a lack of social support and to increase parenting stress [9]. Even when parents find it difficult to build relationships on their own, face-to-face contact with visitors can help prevent social isolation by facilitating connections with neighbors and the local community. As home visit programs provide opportunities to identify parents’ circumstances, including loneliness [10], and link them with social support, their role is likely to become increasingly important [2].

This study has several limitations. First, because existing program evaluation data were used, detailed participant characteristics could not be assessed, and in-depth analyses were not possible. Second, the analysis relied on single-year survey data from one municipality, with a response rate below 40%, which limits generalizability. Respondents might have been somewhat more engaged with the program than nonrespondents, which could have influenced the overall pattern of responses. Further epidemiological studies in Japan are needed to examine the impact of home visit programs on parents’ social connectedness [11].
